# The Effectiveness and Implementation of Psychological First Aid as a Therapeutic Intervention After Trauma: An Integrative Review

**DOI:** 10.1177/15248380231221492

**Published:** 2024-01-28

**Authors:** Ling Wang, Ian Norman, Victoria Edleston, Christopher Oyo, Mary Leamy

**Affiliations:** 1 King’s College London, UK

**Keywords:** mental health and violence, PTSD, trauma and stress, intervention/treatment, effectiveness, implementation

## Abstract

Psychological First Aid (PFA) is known to be an initial early intervention following traumatic exposure, yet little is known about its optimal implementation and effectiveness. This review aims to examine the evidence for the effectiveness of PFA interventions and how PFA interventions have been designed, implemented, and experienced. MEDLINE, the Cumulative Index to Nursing and Allied Health Literature (CINAHL), Cochrane Library, PsychINFO, Embase, Web of Science, PILOTS, and China National Knowledge Infrastructure (in Chinese) databases were searched. Twenty studies from 4,735 records were included and quality rated, followed by an integrative synthesis of quantitative and qualitative evidence. PFA intervention following trauma exposure shows a positive effect for reducing anxiety and facilitating adaptive functioning in the immediate and intermediate term, yet the evidence for reducing Post-traumatic stress disorder/depressive symptoms is less compelling. Furthermore, commonalities in the components and techniques across different PFA approaches identified tend to align with four of Hobfoll’s five essential elements: safety, calm, efficacy, and connectedness (as reflected among 7/11 PFA protocols), whereas the “hope” element was less developed. These commonalities include active listening, relaxation/stabilization, problem-solving/practical assistance, and social connection/referral. Intensive techniques such as cognitive reconstruction have also been incorporated, intensifying PFA delivery. The substantial variation observed in PFA format, timing, and duration, coupled with inadequate documentation of fidelity of implementation and adaptation, further constrains the ability to inform best practices for PFA. This is concerning for lay frontline providers, vital in early trauma response, who report implementation challenges despite valuing PFA as a time-sensitive, supportive, and practical approach.

## Introduction

A traumatic event is defined as an event experienced, witnessed, or confronted by an individual that involves actual or threatened death, serious injury, or a threat to the physical integrity of self or others (American Psychiatric Association, 2013). Exposure to traumatic events such as natural disasters, serious illness, and violence is highly frequent, with epidemiological data indicating that at least 70% of the general population may have been exposed to a traumatic event, with an average of 3.2 lifetime traumas during their lifetime (Benjet et al., 2016; Kessler et al., 2017). While many individuals can adapt to the aftermath of such common traumatic exposure, some may develop enduring difficulties, leading to a broad range of adverse outcomes such as anxiety, depression, substance use, and other function impairments (Atwoli et al., 2015). Post-traumatic stress disorder (PTSD) is the most prevalent psycho-pathological consequence of exposure to traumatic events, with a lifetime prevalence ranging from 1.3% to 22.8% (Karam et al., 2014). This places a significant disease burden as a result of increases in levels of disability, unemployment, and premature mortality (Miller & Sadeh, 2014). There is a societal commitment to prevention and early intervention to address the high prevalence of traumatic exposure and the associated burden (Dyregrov & Regel, 2012). However, despite significant efforts to develop preventative strategies and treatments, the tasks are challenging and complicated given the complexity of identifying high-risk individuals, providing suitable treatments, and as well as addressing significant barriers such as stigma to individuals seeking acute care (Bisson et al., 2021; Roberts et al., 2019). There is now a tendency to focus on the first step: providing immediate early interventions to facilitate adaptation to trauma before any symptoms of PTSD develop.

Psychological First Aid (PFA) is known to be an initial psychosocial support approach to help people affected in the aftermath of trauma exposure, involving the provision of information, comfort, practical assistance, and referral to specialist services if necessary (Ruzek et al., 2007; Vernberg et al., 2008). Originating as a response to managing soldiers’ psychological distress during World War II (Blain et al., 1945), early iterations of PFA have progressed relatively slowly. An increased awareness of the dire consequences of disasters and terrorism, most notably after the 9/11 attacks in the USA, accelerated a renewed interest in alternatives to psychological debriefing, leading to a proliferation of the use of PFA. More recent PFA approaches have been refined to avoid potentially unhelpful elements, that is, emotional catharsis (Shultz & Forbes, 2014). Recent advancements in PFA have embraced a non-prescriptive package rooted in the five essential elements derived from trauma and disaster recovery literature and expert consensus, including the promotion of safety, calmness, self-efficacy, connectedness, and hope (Hobfoll et al., 2007).

Despite the limited direct empirical evidence, the widespread use of PFA to mitigate the adverse impacts of disasters and extreme traumatic events remains undeterred, and numerous PFA models and frameworks have been developed. Collaboration between international organizations and governments has focused on promoting PFA as the minimum intervention approach following mass trauma. Illustratively, the Inter-agency Standing Committee (IASC) guideline on Mental Health and Psychosocial Support (MHPSS) in humanitarian settings makes a clear reference to PFA (Inter-Agency Standing Committee, 2007); while PFA implementation guidelines have also been created to facilitate mental health responses to specific public health emergencies such as the Ebola outbreak and the COVID-19 pandemic response (World Health Organization, 2014). The thriving adoption of PFA can be attributed to its widely accepted strengths, including its simplicity, allowing it to be taught with minimal clinical knowledge that enables broad frontline non-specialist providers without mental health backgrounds to deliver timely support, as well as its flexibility that can be tailored to individuals’ needs. The inherent strength has made PFA a preferred approach, especially as the focus on providing an initial response helps rather than pathologizing individuals for those exposed to various traumatic events. For example, the application of PFA has been extended to supporting individuals experiencing physical injury, homelessness, and crime (Frankova et al., 2022; Shah et al., 2020; Varma et al., 2022). Current consensus continues to endorse the delivery of PFA as the first step in supporting individuals in the aftermath of experiencing traumatic events while acknowledging the need to develop more effective formal preventative interventions (Everly & Lating, 2021; NICE, 2018).

However, the lack of evidence regarding the implementation and effects of a PFA intervention remains a critical concern. A key challenge in the field lies in implementation, including the multitude of PFA models, intervention protocols, program goals, and expected outcomes, with as yet no consensus (Shultz & Forbes, 2014). Meanwhile, the inherent complexities associated with conducting research within disaster and mass trauma contexts have further compounded this challenge, often prioritizing practicality over rigorous research design and outcome evaluation (Church of Sweden, 2018). Previous reviews of PFA commissioned by WHO and the American Red Cross have concluded that there is “no direct evidence to make any clinical recommendations” (Bisson & Lewis, 2009; Dieltjens et al., 2014; Fox et al., 2012), with a recently published review coming to the same conclusion (Hermosilla et al., 2023). Others attempt to summarize evidence for PFA training effectiveness (Movahed et al., 2023; Wang et al., 2021), yet all these endeavors have yet to establish a comprehensive understanding of the PFA intervention itself.

Beyond the imperative to strengthen the evidence base on the effectiveness of PFA, there is also a need to understand how it can best be implemented. First, it is crucial to specify the mechanisms of action for any mental health intervention to demonstrate how it may achieve its desired outcomes, whereas this has rarely been examined for PFA interventions. The extent to which different PFA interventions incorporate the “five essential elements” and their theoretical mechanisms for achieving these desired outcomes is poorly understood. Second, it is important to identify certain PFA intervention components and examine how common intervention characteristics, such as shared goals (principles addressed) and components (techniques applied) that could contribute toward the overall effectiveness of PFA intervention. Third, conducting implementation analysis that explores PFA as the intervention itself, coupled with experiential insights from both recipients and providers, holds the potential to shed light on the dynamics of PFA intervention as it is used in real-world settings. This review has sought to address these knowledge gaps and answer the following research questions:

i. What current evidence exists for PFA effectiveness as a therapeutic intervention?
ii. How have Hobfolls’ principles been operationalized into components of successful PFA interventions?
iii. How have PFA interventions been implemented in terms of population, delivery, format, and setting?
iv. What is the experience and acceptability of PFA among people receiving and delivering it?

## Method

The purpose of this integrative review was to consider all empirical data relevant to the research questions and to generate a theoretical and practical knowledge base for PFA (Whittemore & Knafl, 2005). The integrative review method involved five steps, namely problem identification, literature search, data evaluation and analysis, and result presentation. Reporting was guided by the Preferred Reporting Items for Systematic reviews and Meta-Analysis (Moher et al., 2015).

### Search Strategy

**Figure 1.**
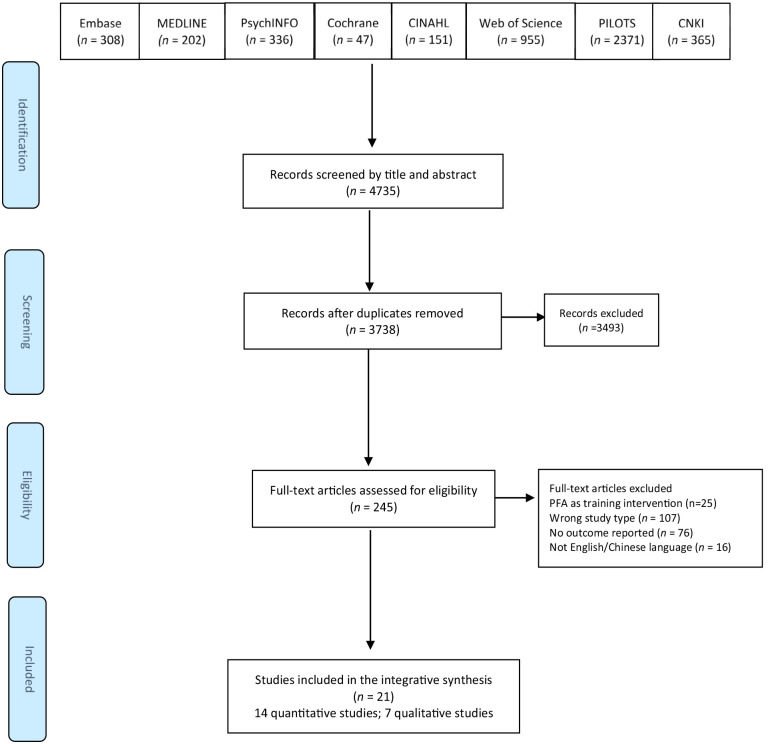
Preferred reporting items for systematic reviews and meta-analysis flowchart.

The search strategy was developed and tested based on an initial literature scan via the MEDLINE database in consultation with the university librarian and review team. Given the scarcity of PFA evidence, we used the keywords “Psychological First Aid” and “PFA” with no Boolean limiters (e.g., for outcomes or design) and no date limit to ensure a sensitive search strategy which would identify a very high proportion of relevant literature. Eight databases, including MEDLINE, the Cumulative Index to Nursing and Allied Health Literature (CINAHL), Cochrane Library, PsychINFO, Embase, Web of Science, PILOTS, and China National Knowledge Infrastructure (in Chinese), were searched (see Figure 1). References of the included papers were hand-searched, and the gray literature was considered through contacting experts in the topic area, using Google and Google Scholar search engines, and searching relevant organizational websites such as the World Health Organization. An initial search was run in December 2019, and the final search was re-run in May 2023.

### Selection Criteria

Considering the varied uses of PFA, this review considered the following two aspects to help define the scope and range of PFA offered. First, given the varied definitions of PFA, for the purposes of this review, we used the WHO definition: “a humane, supportive and practical approach to people suffering severe stressors following trauma exposure and who may need help” (WHO, 2011). Secondly, for the purposes of study selection and analysis, this review further considered the use of “Psychological First Aid” as a structured therapeutic intervention approach underpinned by Hobfoll’s five essential intervention principles (safety, calmness, self-and community efficacy, connectedness, and hope) (Hobfoll et al., 2007), with the aim to reduce the initial distress caused by traumatic events, and to foster short-and long-term adaptive functioning (Brymer et al., 2006). Thus, specific eligibility criteria were developed by the research team and guided by the Sample, Phenomenon of Interest, Design, Evaluation, and Research type framework (Cooke et al., 2012):

- *Sample.* Individuals exposed to a traumatic event who received PFA and/or providers of PFA.
- *Phenomenon of Interest*. An evaluation study of an intervention where PFA was the main intervention focus (or else a constituent practice element of the intervention protocol) and based on Hobfoll’s five essential elements.
- *Design*. Randomized controlled trials (RCTs) or quasi-experimental studies that evaluated PFA interventions and/or studies using any method to assess experiences of providing or receiving PFA.
- *Evaluation*. Any intervention evaluation, including effectiveness outcomes relevant to research questions and experiential accounts of PFA from recipients or providers.
- *Research type*. All types of quantitative, qualitative, and mixed methods studies.
- This review excluded studies which used PFA-based public health strategies to address a mental health crisis at a population level, for example, using psycho-educational messages through the media to non-specific populations.

### Data Extraction

A total of 3,738 articles were obtained from eight databases and uploaded to the review management software Rayyan (Ouzzani et al., 2016). After eliminating duplicates, three team members (LW, ML, and IJN) independently screened all titles and abstracts. Disagreements and uncertainties were resolved through discussion, leading to the exclusion of 3,493 articles and the identification of 245 full-text papers. Articles written in languages other than English or Chinese were excluded. A total of 21 primary research studies were chosen for final inclusion, as shown in Figure 1.

### Data Quality Appraisal

The critical appraisal was conducted by LW independently and discussed with co-authors (IJN and ML). The revised Cochrane risk of bias tool for randomized trials (RoB2) was used to assess the eight RCTs (Sterne et al., 2019). The risk of bias in non-randomized studies-of Interventions (ROBINS-I) tool was used to assess the remaining six non-randomized single-cohort studies (Sterne et al., 2016). The Critical Appraisal Skills Programme (CASP) checklists were used to assess six qualitative research studies (CASP, 2018). Studies were not excluded based on quality ratings, but the ratings were taken into consideration during the data analysis and synthesis stages when deciding how much weight to put on the findings of individual studies.

### Effectiveness Ratings

The results of the eight RCTs, which compared PFA with an evidence or non-evidenced based intervention, were further classified based on the “PracticeWise” evidence hierarchy for mental health intervention trials (https://www.practiseweb.com). Hence, when the PFA intervention performed better than one or more comparisons at the first post-intervention measurement endpoint (regardless of the control intervention), a “win” was recorded for the study. A “valid tie” was recorded if a PFA achieved a similar effect on a given outcome as other evidence-based comparisons. A “non-valid tie” was recorded for an equivalent effect between PFA and a non-evidence-based intervention. Finally, a “loss” was recorded when PFA performed worse than any comparison intervention.

### Data Synthesis

Parallel narrative syntheses to address each of the four research objectives were conducted (Patton, 2014). For objective 1, study characteristics and PFA outcomes were tabulated to assess effectiveness and implement the practicewise classification (Table 1). For objective 2, the common components of each intervention were identified, charted, and compared to Hobfoll’s five essential elements (safety, calming, self-efficacy, connectedness, and hope). For objective 3, the key characteristics of the intervention were charted using the template for intervention description and replication tool (TIDieR) (Hoffmann et al., 2014) (Table 2). For objective 4, data on providers’ and recipients’ experiences of PFA interventions were extracted and synthesized (Table 3).

**Table 1.** Summary of 21 Included Studies Investigating PFA Interventions.

Study		Participants		Intervention				Measure	Outcomes				
Author	Design	Participants and Conditions	Types of Trauma	Content and Theoretical Framework	Modality	Duration	Provider	Timepoint	PTSD Symptom	Depressive Symptom	Anxiety	Resilience	Functioning
Figueroa et al. (2022) Chile	RCT	221 adult patients *n* = 109 PFA *n* = 112 psychoeducation	An actual or threatened death, serious injury, witnesses others’ trauma experience	ABCDE PFA: active listening, breathing retaining, needs categorization, referral, psychoeducation	Individual Face-to-face	1 1 hr session	Non-specialist	Baseline 1 month 6 month	✓	✓	✓	—	—
									PCL-S	BDI-II (1 month only)	VAS (immediate only)	—	—
									Win (Fewer PTSD symptom ↓: *p* = .33) only at 1 month; Nonvalid tie (no change in depressive symptom—: *p* = .713) at 1 month; Win (greater distress relief ↓: *p* = .38) immediately after PFA; No difference in the frequency of PTSD at 1 month (*p* = .408).				
Ramirez et al. (2021) U.S.A.	RCT	314 children in age 14 *n* = 155 PFA *n* = 159 trauma education	An unintentional injury	Link for Injured Kids: integrated with motivational interview and stress-screening	Individual Face-to-face	N/A	Non-specialist	Baseline 6 week 3 month 6 month	✓	✓	✓	—	✓
									CPSS	CES-D	K-6	—	PedsQL; SDQ
									Nonvalid tie in all outcomes (both improved symptoms of posttraumatic stress, depression, and pediatric quality of life at similar rates over time); PFA group had additional improvements in emotional behavior, conduct, and peer behaviors.				
Ironson et al. (2021) USA	RCT	105 community residents *n* = 34 PFA *n* = 34 EMDR *n* = 37 SMT	Crime community	Selected NCTSN PFA: Stabilization, safety and comfort, coping linkage with collaborative services	Group Face-to-face	4 90 min–2 hr session 4 weeks	Specialist	Baseline 1 month 3 month 6 month	✓	✓	—	—	—
									Davidson PTSD; PTCI	BDI-II	—	—	—
									Valid tie in all outcomes (all achieved significant declines in PTSD symptom over time, effect size *d* = 0.96 at 1 month; *d* = 1.38 at 3 month; *d* = 1.10 at 6 month; as well as BDI symptoms *d* = 0.71); EMDR with the fastest declines, followed by SMT and PFA.				
McCart et al. (2020) USA	RCT	172 individuals *n* = 79 PFA *n* = 93 usual care	Rape, sexual or physical assault, armed robbery, physical injury	NCTSN PFA:8 steps component with victim engagement and stabilization prioritized	Individual Face-to-face	2/3 session	Non-specialist	Baseline 1 month 2 month 4 month	✓	✓	✓	—	✓
									NSEPS	BSI-18	BSI-18	—	ASI-SR; SAS-SR
									Nonvalid tie in NSEPS and BSI (both groups had improvement), whereas female PFA group experienced greater early reduction in depressive symptoms. Win (PFA has better improvement in global functioning: ß = .24, *t* = 2.21, *p* = .03).				
Despeaux et al. (2019) USA	RCT	119 college students *n* = 59 RAPID PFA *n* = 60 group discussion	Watched 5 min video of violence and injuries related to an attack on civilians in Syria	RAPID PFA: establishing rapport, reflective listening, validating response, reframing, self-care	Group Face-to-face	1 session	Specialist	Baseline After video After PFA 30 min	—	—	✓	—	—
									—	—	STAI PANAS	—	—
									Win in all outcomes (STAI ↓: *p* < .001; PA ↑: *p* < .001; NA↓: *p* < .001), PFA group had more significant effect immediately after, and even more so after 30 min delay.				
Biggs et al. (2016) USA	RCT	126 soldiers *n* = 68 PFA *n* = 58 assessment only	Post-deployment after war	TEAM PFA: integrated with psychological recovers, cognitive behavior therapy, battle-mind training, early collaborative care	Group Face-to-face	4 2-hr session 10 months	Specialist	Baseline,1, 2, 3, 4, 7, and 10 months	✓	✓	—	—	✓
									PCL-17	PHQ-9	—	—	WHOQOL-BREF
									Nonvalid in all outcomes (both achieved PTSD & depression symptoms decreased, and QOL improved over 10 months).				
Everly et al. (2016) USA	RCT	42 college students *n* = 19 RAPID PFA *n* = 23 social acknowledgment	Discussed a stressful life event	RAPID PFA: establishing rapport, reflective listening, validating response, reframing, self-care	Individual Face-to-face	1 session	Non-specialist	Before, after, again after 30 min	—	—	✓	—	—
									—	—	STAI POMS	—	—
									Win in STAI (*d* = 0.82) and POMS (*d* = 0.45), only at 30 min post-intervention.				
Meir et al. (2012) Israel	RCT	99 children aged 6 *n* = 52 PFA *n* = 47 drawing	Children threatened with impending deportation	NCTSN PFA: Establishing rapport, Iggy and Ziggy’s activities, fear-producing experience sharing, emotion-stabilization, and coping strategies	Group Face-to-face	1 20–30 min session	Specialist	Baseline 8 week	—	✓	✓	—	✓
									—	BPI	BPI	—	Strategies Basket Test
									Win (Depressive ↓: *p* < .01; Anxiety ↓: *p* < .01); PFA group had lower anxiety and hyperactivity, depression symptoms, and more social acceptance.				
Farchi et al. (2018) Israel	Single cohort	211 individuals	War time crisis	ICF-PFA: Cognitive communication Challenge & control Commitment Continuity	Individual/group Face-to-face	1 45 min session	Non-specialist	Baseline 2 month 4 month	✓	—	✓	✓	—
									Foa’s 17 item PTSD	—	STAI	GSE	—
									↓(*p* < .001)	—	↓ (*p* < .000)	↑ (*p* < .05)	—
Ramiez et al. (2013) USA	Single cohort	20 middle school children aged 14	Great flood, violence, or death of a loved one	Listen Protect Connect: reflective listening, safety, support, facilitate resource/care resource	Individual Face-to-face	1 session	Non-specialist	Baseline 2 week 4 week 8 week	✓	✓	—	✓	✓
									The modified Child PTSD	CES-D	—	HKRMSC	MSPSS
									↓ (*p* = .09)	↓(*p* < 0.01)	—	↑ (*p* < 0.01)	↑ (*p* < 0.01)
Hechanova et al. (2019) Philippine	Single cohort	65 official employees	Fire accident	NCTSN PFA: Introduction, group sharing, mindfulness, problem solving, closing activity	Group Face-to-face	5 sessions 3 days	Specialist	Baseline 1 week	✓	—	—	✓	
									PCL-6	—	—	CDRS	Social support
									As screening	—	—	↑ (*p* < .01)	↑ (*p* < .01)
Hechanova et al. (2015) Philippine	Single cohort	98 local government employees	Typhoon Haiyan	NCTSN PFA: Mindfulness training, group sharing, stress coping, problem-solving, closing activity	Group Face-to-face	N/A	Specialist	Baseline Post PFA	✓	—	—	✓	✓
									PCL-C	—	—	2-item scale	coping scale
									As screening	—	—	↑ (*p* < .01)	↑ (*p* < .01)
Mueller et al. (2011) Philippine	Single cohort	463 adult patients	Displacement in Mindanao conflict	WHOPFA: Psychoeducation, breathing/relaxation exercises, problem-solving, cognitive behavioral techniques	Individual Face-to-face	5 session 6 months–2 years	Specialist	Baseline 1, 2, 3, 4, 5, 6, and 7 visits	—	—	✓	—	✓
									—	—	SRQ20	—	GAF
									—	—	↓ (*p* < .001)	—	↑ (*p* < .001)
Cain et al. (2010) USA	Single cohort	99 children aged 10	Hurricane Katrina	NCTSN PFA: recognizing emotional and coping strategies, self-care, re-enjoy	Group Face-to-face	N/A 6 weeks	Non-specialist	Baseline 1 month	✓	—	—	—	✓
									CPTS-RI	—	—	—	coping scale
									↓ (*p* < .027)	—	—	—	—
Legerski et al. (2012) USA	Focus group	21 disaster relief workers	Hurricane	WHO PFA	Individual/group Face-to-face	N/A	Non-specialist	Major findings					
								• PFA providers: i. Challenges: facing a variety of physical, financial, and mental health needs and also for ongoing secondary adversities; the difficulty of early identification of children in need of mental health care services; lack of social support. ii. Barriers: limited guidance/training on making contact and engaging victims, especially the cultural competence; unmanageable self-care.					
Schafer et al. (2015) Israel	Focus group	8 providers and 23 recipients	War conflict	WHO PFA	Individual Face-to-face	N/A	Non-specialist	• PFA recipients: i. PFA was a generic to social support. ii. Improved psychosocial well-being (improved safety in crisis, enabled calming, improved social connectedness, and greater sense of control and hope, and life functioning); facilitated physical safety. • PFA providers: i. Concerns about pushing to share traumatic memories and feelings while not being able to deal with. ii. Insufficient for meeting the extensive psychosocial needs of the crisis-affected population. iii. Need a robust structure; adapting PFA for context, culture, and gender.					
Horn et al. (2019) West Africa	interview	55 providers	Ebola outbreak	WHO PFA	Individual Face-to-face	N/A	Non-specialist	• PFA providers: i. Endorsed PFA as a useful early intervention after trauma. ii. Concern: challenged by inadequate clear grasp of the PFA itself and need emotional support for themselves; also emphasized general difficulties to maintain the fidelity to the original PFA on each element. iii. Enablers to reduce diluted PFA delivery: small group training; supervision; role-play and receive feedback on their performance; contextualize the materials.					
de Freitas Girardi et al. (2020) Canada	Focus group	Two groups of providers	Refugee	WHO PFA	Group Face-to-face	N/A	Non-specialist	• PFA providers: i. Perceived benefits for recipients: foster emotional safety and comfort; enabling the expression of past or present experiences in a resilience-oriented way; promote self-efficacy in children. ii. Challenges of PFA implementation: lack of material and mental health service resources in shelters; lack of an adapted space often made establishing comfort and safety challenging.					
Blake et al. (2021) UK	Interview	24 health workers	COVID-19 pandemic	WHO PFA	Individual Face-to-face	N/A	Non-specialist	• PFA recipients: i. A value for workplace well-being that staff greatly appreciated and described as “friendly,” “supportive,” and “good at listening,” allowing them to offload and share their worries and concerns. ii. Active listening was the most valued element of their presence; peer- to-peer psychological support is valued, and the buddy role served a dual purpose: skill development and contribution to workforce well-being.					
Tessier et al. (2022) Canada	Interview	13 emergency medical service workers	Exposed to a traumatic event	Peer-help PFA	Individual Face-to-face	N/A	Non-specialist	• PFA recipients: i. A straightforward and early-on intervention that they greatly appreciated its time-sensitive nature and the closeness with peers, felt listened to and respected, surrounded and supported, understood, calmed and reassured, and equipped through PFA intervention. ii. Benefits over time including the reduction of stigma, the increase of help-seeking behaviors, and the decrease of organizational difficulties. • PFA providers: i. Benefits of selecting co-workers as a provider: shared experience of the job eased the partnership and enhanced trust and openness. ii. Indicated potential outcome in long run: fostered demand for and access to psychosocial resources; improved destigmatization of mental health issues; enabled faster and more efficient return to work as an organizational outcome.					
Geoffrion et al. (2023) Canada	Interview	26 police officer	Exposed to a stressful event	NCTSN PFA	Individual Face-to-face	N/A	Non-specialist	• PFA recipients: i. PFA, as a useful, flexible, and helpful tool, offers easy access to psychological support and rapid response to traumatic events. ii. Benefits over time include meeting their long-term needs and being more open to talking about stress, genuine demonstrations of concern for their well-being, create a sense of hope. • PFA providers: i. Benefits of of peer provider enable openness and facilitate second-level care; being a PFA provider is a natural continuation of the supervisors’ role and ensure proactivity. ii. Challenges: being a provider is demanding and raised concerns such as emotional burden to their role; lack of time.					

*Note.*Provider: Specialist: intervention provided by someone with a master-level qualification or above; non-specialist: intervention provided by someone without a masters-level qualification. Modality: Individual: intervention delivered 1:1; Group: intervention delivered in a group. Study outcomes were identified using an established PracticeWise Evidence-Based Service evidence hierarchy. A “win” was applied to a study in which an index intervention performed better than one or more comparison groups at the first post-treatment end point. A “valid tie” was applied to studies where the index intervention achieved a similar effect on a given outcome as one or more other evidence-based interventions. A “nonvalid tie” reflected equivalence between the index intervention and a non-evidence-based protocol. A “loss” occurred when the index intervention performed worse than comparison group.ASI-SR = the Addiction Severity Index-Self-Report; BDI-II = Beck Depression Inventory-II; BSI-18 = the 18-item Brief Symptom Inventory; CDRS = the Connor-Davidson Resilience scale; CES-D = the Center for Epidemiologic Studies Depression scale; CPSS = the Child PTSS Symptom Scale; CPTS-RI = the child post-traumatic stress reaction index; EMDR = Eye Movement Desensitization and Reprocessing; GAF = Global Assessment of Functioning; GSE = the General Self-Efficacy scale; HKRMSC = the Healthy Kids Resilience Measure of School Connectedness; K-6 = the short-form Kessler Psychological Distress Scale; NSEPS = the National Stressful Events PTSD Survey; PANAS = the Positive and Negative Affect Schedules; PCL-17 = the 17-item PTSD Checklist; PCL-S = Posttraumatic Stress Disorder Checklist specific version; PedsQL = the Paediatric Quality of Life Inventory; PHQ-9 = Patient Health Questionnaire-9; POMS:the Brief Profile of Mood States; PSS = the Perceived Stress Scale; PTCI = Post traumatic cognitions inventory; SAS-SR = the Social Adjustment Scale-Self Report; SDQ  = the Strengths and Difficulties Questionnaire; SMT = Stress Management Treatment; SRQ20 = self-reported distress questionnaire; STAI = Spielberger’s State-Trait Anxiety Inventory; VAS = Visual analog scale; WHOQOL-BREF = the 26-item World Health Organization Quality of Life Assessment—Brief Version.

**Table 2.** Summary of 14 PFA Intervention Experimental Studies and Outcomes According to the TIDieR Checklist.

Study	Ix	Outcome				Why	Who				How and Where					When and How Much						Tailoring	How Well		Total
		PTSD/Depressive Symptom	Anxiety	Resilience	Function	Theory	From		To		Screen	Deliver				Duration			Intensity				Attrition	Adherence	
							sp	ns	p	np		ftf	com	ind	gr	Short ≤ 1 day	Med 1 week	Long >1 week	Low ≤1 sessions	Med 2–4 sessions	Long >4 sessions				
Figueroa (2022)	WHO	√	√	—	—	•		•	•		•	•		•		•			•			◉	•	⊘	9
Ironson (2020)	NCTSN	√	—	—	—	⊘	•			•	•	•			•			•		•		◉	•	•	9
McCart (2020)	NCTSN	√	√	—	—	•		•		•	—	•		•				•		•		•	⊘	•	9
Despeaux (2019)	RAPID	—	√	—	—	⊘	•			•	—	•			•	•			•			◉	⊘	⊘	6
Biggs (2016)	NCTSN	√	√	—	√	⊘	•			•	—	•			•		•			•		⊘	•	⊘	7
Everly et al. (2016)	RAPID	—	√	—	—	⊘		•		•	—	•		•		•			•			◉	⊘	⊘	6
Ramirez (2021)	Link	√	√	—	√	⊘		•	•		—	•		•		—			—			◉	•	—	5
Meir (2012)	NCTSN	√	√	—	√	•	•			•	—	•			•	•			•			•	⊘	⊘	8
Farchi (2018)	SIX Cs	√	√	√	—	•		•	•		—	•		•		•			•			•	⊘	⊘	8
Ramirez (2013)	Link	√	—	√	√	•		•		•	—	•		•		•			•			•	•	⊘	9
Hechanova (2019)	NCTSN	—	—	√	√	•	•		•		•	•			•		•				•	•	⊘	⊘	9
Hechanova (2015)	NCTSN	—			—			√	√	•	•			•	•	•			•	—	—	•	⊘	⊘	7
Yolanda (2011)	WHO	—	√	—	√	⊘	•		•		—	•		•				•	—			⊘	•	⊘	6
Cain (2010)	NCTSN	√	—	—	√	•	•			•	—	•			•			•	—			⊘	⊘	⊘	6
Total		8	9	4	8	8	8	6	5	9	4	14	0	7	8	6	2	4	6	3	1	6	6	2	

*Note.* √ = effect; ⊘ = not reported; ◉ = adjunct to main PFA intervention model; • = reported; — = not applicable; d = day; ftf = face-to-face only; com: combination, for example, face-to-face, supervision, etc.; gr = group; ind = individual; Ix = PFA therapeutic intervention model; med = medium; np = outpatient individuals, including crime victims, conflict-affected individuals, etc.; ns = non-specialist including health workers, community worker, and social workers etc.; p = patients including hospital and clinical patients; se = sessions; sp = specialist including psychologist, psychiatrist, mental health researchers with master degrees; TIDieR = template for intervention description and replication; w = week; X = no effect.

**Table 3.** Critical findings from this integrative review of PFA.

Issue	Findings
PFA effectiveness evidence	• PFA evaluations have focused on assessing its effect on traumatic stress symptoms and adaptive functioning, with some promising immediate and intermediate outcomes. • Randomized controlled trials assessing PTSD/depressive symptom outcomes have shown a small but significant positive effect that warrants replication. • Anxiety, the most frequently assessed outcome indicator, has shown an overall significant reduction effect across all study designs. • Adaptive functioning outcomes, assessed using quality of life and coping measures, have shown positive improvement effects in trials. Resilience and self-efficacy were not assessed extensively but have also produced significant improvement in pre-post studies when PFA was delivered in mass trauma.
Intervention component commonalities	• Current PFA interventions tend to incorporate four of Hobfoll’s five essential elements, including safety, calm, efficacy, and connectedness (as reflected among 7/11 PFA protocols), whereas “hope” element was less developed. • These commonalities include active listening, relaxation/stabilization, problem-solving/practical assistance, and social connection/referral. • Intensive techniques such as cognitive reconstruction have also been incorporated, intensifying PFA delivery.
Implementing in trauma context	• All evaluated PFA interventions are delivered in a face-to-face format, either individually or in groups. • The timing of delivery varies from immediately following trauma exposure to up to 2 years later, with varying intensity ranging from one single session to multiple sessions lasting from 2 weeks to 10 months. • Shorter single-session PFA is typically delivered by non-specialist providers, while mental health specialists mainly deliver more intensive multi-session PFA. • Inadequate documentation on intervention protocols, particularly regarding adaptation and fidelity, results in poor clarity for PFA implementation.
Experience with receiving or providing PFA	• Both recipients and providers perceive PFA as a time-sensitive, supportive, and practical approach to facilitate symptomatic and functional outcomes following trauma exposure. • Peer provider and group formats are highly valued in interdependent cultures and organizational contexts, showing longer-term benefits, including reduced stigma, increased help-seeking behavior, and decreased organizational difficulties. • Lay providers have expressed concerns about inadequate training and adaptation of PFA, which could lead to difficulties establishing rapport with the recipient, confusion about the intervention structure, emotional burden, and harmful delivery.

*Note.* PFA = Psychological First Aid; PTSD = Post-traumatic stress disorder.

## Results

### Study Characteristics

Among the 21 included studies, 8 quantitative studies were RCTs, 6 were single cohort studies and 7 more were qualitative studies. Around half were conducted within the past 5 years (*n* = 11) and were undertaken in the American continent (*n* = 13), followed by Asia (*n* = 6), Africa (*n* = 1) and Europe (*n* = 1). Seven qualitative studies used semi-structured interviews and focus groups, and four of the quantitative studies employed post-intervention open-question surveys to assess provider/recipient views. The type of trauma experienced by individuals in the studies included: mass trauma (e.g., natural disasters and fire accidents) (*n* = 6), secondary trauma (e.g., witnessing others’ trauma experience) (*n* = 4), injuries (e.g., car accidents and physical injuries) (*n* = 3), interpersonal violence (e.g., sexual abuse and rape) (*n* = 3), collective violence (e.g., war, ongoing conflicts, and displacement) (*n* = 4), and death of a loved one (*n* = 1).

Most studies sampled adult populations, apart from four studies that comprised only children (mean age = 10) and two studies of college students (age range from 18 to 21). The number of traumatized individuals randomized to the eight trials ranged from 42 to 314 (mean = 147), with six trials including sample sizes of over 100. Conversely, among the six single-cohort studies, only one study included 463 participants; the other five studies recruited smaller samples from 20 to 98.

Studies employed well-validated measures to assess PTSD symptoms (*n* = 8), anxiety (*n* = 8), depression (*n* = 7), resilience (*n* = 3), self-efficacy (*n* = 2), adaptive functioning (*n* = 2), coping (*n* = 2), quality of life (*n* = 2), social support (*n* = 2) and substance use (*n* = 1); while four studies employed “ad hoc” measurement tools to assess resilience and functioning. Given the significant heterogeneity in the selection processes, assessment tools, and follow-up times across these studies, a meta-analysis to pool the findings was inappropriate.

### Quality Appraisal

The critical appraisal of quality ratings showed that the eight RCTs were at moderate to high risk of bias (Biggs et al., 2016; Despeaux et al., 2019; Everly et al., 2016; Figueroa et al., 2022; Ironson et al., 2021; McCart et al., 2020; Meir et al., 2012; Ramirez et al., 2021), due to significant differences of the baseline group characteristics (Biggs et al., 2016), inadequate reporting on the randomization process and blinding (Biggs et al., 2016; Despeaux et al., 2019; Ironson et al., 2021; Meir et al., 2012), deviation from the PFA intervention protocol (Ironson et al., 2021; McCart et al., 2020) and incomplete analysis of outcome data (Figueroa et al., 2022; Meir et al., 2012; Ramirez et al., 2021). Of the six single cohort intervention studies, four were assessed as having a moderate risk of bias due to poor adjustment of confounding variables, deviations from planned interventions, and non-validated measurement of outcomes (Farchi et al., 2018; Hechanova et al., 2015, 2019; Ramirez et al., 2013). Two more were found to have a serious risk of bias due to deviation from the intervention protocol and the use of non-valid measurement tools (Cain et al., 2010; Mueller et al., 2011). The quality of the seven qualitative studies varied, with limitations including lack of researcher reflexivity (Blake et al., 2021; de Freitas Girardi et al., 2020; Geoffrion et al., 2023; Horn et al., 2019; Tessier et al., 2022), use of convenience sampling (Legerski et al., 2012; Schafer et al., 2015) and failure to use a systematic method of data analysis (Legerski et al., 2012).

### Synthesis 1: Effectiveness of PFA Interventions

The effectiveness evaluations centered on changes in symptomatology, well-being, and specific functioning outcomes due to the overarching aim of PFA to reduce initial distress from exposure to traumatic events and facilitate adaptive functioning. Most studies focused on immediate and intermediary outcomes spanning post-intervention up to a 6-month duration.

#### PTSD Symptoms

Five trials measured PTSD symptoms as a primary outcome. One trial obtained a “win,” with PFA performing better than psycho-education at 1 month (Figueroa et al., 2022). Another trial achieved a “valid tie” with the PFA intervention being equivalent to other evidence-based interventions (Eye Movement Desensitization and Reprocessing and Stress Management Treatment) at reducing PTSD symptoms and post-traumatic cognitions (effect size *d* = 0.96 at 1 month) (Ironson et al., 2021). One trial obtained a “nonvalid tie” with the PFA group achieving similar non-significant PTSD symptom reduction compared with a non-evidence-based control group (trauma education and assessment only) (Biggs et al., 2016). Similarly, one trial also obtained a “nonvalid tie,” but both groups showed no improvement in psychiatric symptoms (McCart et al., 2020). Moreover, the most robust trial, Figueroa et al. (2022) using a clinician-administrated diagnosis of PTSD, reported a non-significant effect on PTSD symptom frequency after 1 month of intervention (*p* = .408).

Five trials conducted a follow-up assessment at 3 and 6 months post-intervention (Biggs et al., 2016; Figueroa et al., 2022; Ironson et al., 2021; McCart et al., 2020; Ramirez et al., 2021). Only one trial (Ironson et al., 2021) found a reduction in PTSD symptoms sustained at 3 and 6-month follow-ups, with large pre-post effect sizes (3 months: *d* = 1.38, 6 months: *d* = 1.10). In addition, three single cohort studies which measured PTSD symptoms pre and post-PFA, showed a statistically significant reduction in symptoms during follow-ups at 1 to 4 months (Cain et al., 2010; Farchi et al., 2018; Ramirez et al., 2013).

#### Depressive Symptoms

Six trials measured depressive symptoms using self-report measures (Biggs et al., 2016; Figueroa et al., 2022; Ironson et al., 2021; McCart et al., 2020; Meir et al., 2012; Ramirez et al., 2021). Two were classified as a “win” or “valid tie,” with the PFA group reporting a significant reduction of depressive symptoms at the post-intervention endpoint (Ironson et al., 2021; Meir et al., 2012). The four remaining trials obtained a “nonvalid tie.” Two reported no change in depressive symptoms compared to psycho-education and usual care at 1 month (Figueroa et al., 2022; McCart et al., 2020), but two further trials showed that PFA achieved a similar non-significant reduction of depressive symptoms compared to assessment control groups at first post-intervention time-point (Biggs et al., 2016; Ramirez et al., 2021). At 3 and 6-month follow-ups, two trials reported a significant reduction in depressive symptoms for both groups (Biggs et al., 2016; Ironson et al., 2021). One single-cohort study had a significant difference in depressive symptom reduction at all follow-ups compared to baseline measures (Ramirez et al., 2013).

#### Anxiety

Eight studies evaluated anxiety as a critical outcome (Despeaux et al., 2019; Everly et al., 2016; Farchi et al., 2018; Figueroa et al., 2022; McCart et al., 2020; Meir et al., 2012; Mueller et al., 2011; Ramirez et al., 2021). Among six trials that evaluated anxiety levels in general trauma contexts, four obtained a “win” at all time points as PFA produced superior anxiety reduction compared to the control group regardless of intervention type (Despeaux et al., 2019; Everly et al., 2016; Figueroa et al., 2022; Meir et al., 2012). The other two studies achieved a “nonvalid tie” in that PFA also achieved similar reduction in anxiety when compared with trauma education and usual care (McCart et al., 2020; Ramirez et al., 2021). Two single cohort studies of adults during the Israel war and patients displaced in the Philippine conflict also found a significant difference in anxiety reduction after receiving the PFA intervention (Farchi et al., 2018; Mueller et al., 2011).

#### Resilience and Self-efficacy

No trials reported outcomes related to resilience and self-efficacy. However, four studies, two single cohort studies evaluating self-efficacy immediately after PFA intervention and 4 month (Farchi et al., 2018; Hechanova et al., 2015) and two evaluating resilience 1 week after intervention and 8 weeks (Hechanova et al., 2019; Ramirez et al., 2013) showed significant improvements in these outcomes using validated instruments.

#### General Functioning

Nine studies explored other functional outcomes, including quality of life, coping, social support, and substance use. Three trials included a validated measure of quality of life. One obtained a “win,” indicating PFA outperformed usual care in improving adaptive functioning (McCart et al., 2020); the other two trials obtained a “non-valid tie,” suggesting that PFA produced a similar improvement in social behavior and quality of life over time as the other trialed interventions (Biggs et al., 2016; Ramirez et al., 2021). One trial found more coping and acceptance in children receiving a PFA intervention after 8 weeks (Meir et al., 2012). Additionally, four single cohort studies found that perceived approaches to coping with stressful situations, social support, or social adjustment were significantly improved after the PFA intervention (Hechanova et al., 2015, 2019; Mueller et al., 2011; Ramirez et al., 2013).

### Synthesis 2: PFA Components Commonalities or Techniques in Relation to Five Hobfoll’s Essential Elements

An analysis was undertaken of the available protocols from the eleven quantitative studies that produced positive change outcomes to determine how Hobfoll’s five essential elements had been operationalized in these PFA interventions.

#### Principle 1: Promote A Sense of Safety

Promoting a sense of safety refers to first ensuring an individual is in a safe place in order to interrupt the traumatic stimulus (Hobfoll et al., 2007). In practice, the principle of safety was not typically emphasized as a standalone component in PFA protocols. Instead, providers were just given guidance on the need to give space to individuals to express their concerns in a non-directive manner and ensure their voice was heard. Techniques emphasized by most protocols included “rapport building” (Despeaux et al., 2019; Everly et al., 2016), “active listening” (Ramirez et al., 2013, 2021), “contact and engagement” (McCart et al., 2020), “safety and comfort” (Meir et al., 2012), and “cognitive communication” (Farchi et al., 2018). Some protocols contained information on how to develop a safety plan for suicide prevention (McCart et al., 2020).

#### Principle 2: Promote Calm

Promoting calm aims to target agitation through calming extreme emotions and responding to immediate concerns (Hobfoll et al., 2007). In practice, “therapeutic grounding” techniques were adopted based on the person and their specific situation. This component was fully operationalized in most PFA protocols and was often described as a “needs assessment” (Despeaux et al., 2019; Everly et al., 2016; Figueroa et al., 2022; McCart et al., 2020), “control” (Farchi et al., 2018), or “stabilization” (McCart et al., 2020; Meir et al., 2012). The three most frequently used techniques identified were breathing retraining, progressive muscle relaxation, and mindfulness practice. Structured screening was sometimes used to assess immediate concerns or needs (McCart et al., 2020). Normalizing stress reactions and promoting problem-focused coping are also vital techniques to enhance calmness, yet only a few protocols mentioned it as a section or provided information on this (Figueroa et al., 2022). Notably, the “RAPID PFA” model described two steps in their protocol that comprised “Assessment” (i.e., assessing need by listening) and “Prioritisation” (i.e., a form of psychological triage which prioritizes the urgent need to intervene) (Despeaux et al., 2019; Everly et al., 2016). The techniques used to foster principle 2 also contributed to reducing anxiety and overlapped with increasing a sense of safety and promoting hope in accordance with principles 1 and 5 (Hechanova et al., 2015; McCart et al., 2020; Ramirez et al., 2021).

#### Principle 3: Promote Self and Collective Efficacy

Building self-efficacy is critical to recovery after trauma exposure to help individuals regain control over their abilities and skills. This principle has been fully developed in PFA protocols. Main approaches targeted (a) boosting self-efficacy and (b) setting achievable goals for current problems. Protocols described the use of “adaptive coping” (Hechanova et al., 2015, 2019; Meir et al., 2012), “problem-solving” (Hechanova et al., 2015, 2019), and “practical assistance” (Meir et al., 2012). Some protocols highlighted the use of techniques to validate positive coping strategies, educate on goal-oriented thinking, and set achievable goals (Farchi et al., 2018; Figueroa et al., 2022). Among these, promoting the use of existing internal resources (i.e., beliefs) and external resources (i.e., social support) and encouraging group sharing of joint concerns allowed for a sense of collective efficacy to emerge. Techniques such as cognitive behavior therapy (CBT) were also used to reconstruct an individual’s perception of a trauma event and recap successful coping strategies (Despeaux et al., 2019; Everly et al., 2016; Meir et al., 2012).

#### Principle 4: Promote Connectedness

Connectedness is essential for individuals who have experienced trauma and may feel unable to seek help. PFA protocols emphasized this as a key component, using terminology such as “connection with social support” (Farchi et al., 2018; McCart et al., 2020; Meir et al., 2012; Ramirez et al., 2021), “referral” (Figueroa et al., 2022), and “disposition” (Despeaux et al., 2019; Everly et al., 2016). The protocols often included encouraging individuals to connect with loved ones and seek social support. This guidance included ensuring that families or friends were kept together, connecting with spiritual communities, and encouraging people to re-engage in activities that were previously a source of enjoyment (Figueroa et al., 2022; McCart et al., 2020; Ramirez et al., 2013, 2021). Sometimes, social support brochures providing resources were offered (Figueroa et al., 2022). Individuals who experienced severe reactions after trauma exposure were sometimes referred or guided toward professional, social, or clinical services (McCart et al., 2020). The techniques suggested in this component could also promote collective efficacy and overlap with principle 3.

#### Principle 5: Promote Hope

Promoting hope refers to the practice of combating self-defeating thoughts and fostering positive behaviors. These can be addressed by a broad range of techniques, including normalizing trauma reactions and using treatment therapy such as CBT. Some protocols explicitly used cognitive restructuring dialog to “de-catastrophize” negative thoughts (Despeaux et al., 2019; Everly et al., 2016). Other techniques included group singing of inspirational songs (Hechanova et al., 2015, 2019) and motivational interviewing to help injured children find their intrinsic strength to foster positive behavior change (Ramirez et al., 2021). However, techniques for fostering hope were less frequently applied in most protocols than in the other four principles.

### Synthesis 3: PFA Intervention Implementation

Data from all interventions were extracted using the TIDieR tool (Table 2) to map how have PFA interventions been implemented in terms of delivery, format, and setting.

#### Theory: Theoretical Basis of PFA Intervention Components

Despite the importance of having a theoretical basis, many reviewed studies simply cited that reputable PFA guidelines had been followed without providing detailed protocol descriptions. Seven studies implemented the PFA model from the “National Child Center Traumatic Stress Network” including the eight-step protocol, but none articulated the theoretical underpinnings of the model or explained why specific components were included (Biggs et al., 2016; Cain et al., 2010; Hechanova et al., 2015, 2019; Ironson et al., 2021; McCart et al., 2020; Meir et al., 2012). Only one paper implementing a PFA model designed by the World Health Organization articulated its rationale for modifying it to produce a five-step protocol (Figueroa et al., 2022). One intervention which was informed by theory, was grounded in the Immediate Cognitive Function PFA model described by Farchi et al. (2018) and integrated Hobfoll’s five elements with a neurobiological theory of stress and resilience.

#### Who: PFA Intervention Provider

The providers of PFA interventions varied depending on the context of the trauma and the specific goals of the intervention. In general, mental health specialists were the main providers for treating dysfunctional adaptation after trauma exposure (Biggs et al., 2016; Cain et al., 2010; Despeaux et al., 2019; Hechanova et al., 2015, 2019; Ironson et al., 2021; Meir et al., 2012; Mueller et al., 2011). However, in areas such as personal trauma, non-specialist providers such as social workers, school nurses, and law enforcement volunteers were commonly employed (Everly et al., 2016; Figueroa et al., 2022; McCart et al., 2020; Ramirez et al., 2013, 2021). The choice of non-specialist providers was influenced by limited budgets, convenience, and preference for local workers or those closer to the affected individual. For example, Ramirez et al. (2021) prepared parents of children who had physical injuries to deliver PFA at home once the child was discharged from the emergency department.

#### How and Where: PFA Delivery Format and Setting

All PFA interventions were delivered face-to-face. Two also used telephone or the internet for screening, resource dissemination, and follow-up assessments (Figueroa et al., 2022; McCart et al., 2020). An equal number of studies used individual and group delivery methods. Most were delivered in a safe location near where the mass trauma had occurred, such as an emergency health center or school. When delivering PFA to those exposed to a general trauma like a physical injury or crime, the chosen location was often where they sought medical treatment (Figueroa et al., 2022) or where law enforcement officials reported the case (McCart et al., 2020).

#### When and How Much: PFA Intervention Schedule and Intensity

**Figure 2.**
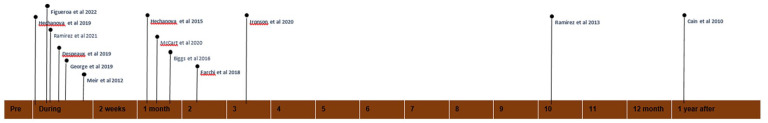
Timeline, in months, of Psychological First Aid intervention delivery. *Note.* Intervention studies (*n* = 13) describing interventions with reliable change in outcomes are indicated in bold.

Thirteen studies reported how long the PFA intervention had been delivered after the trauma event (see Figure 2). Six delivered PFA during and 2 weeks after trauma exposure, and five more after 1 month. Two studies delivered PFA at 10 months and 1 year after the traumatic event (Cain et al., 2010; Ramirez et al., 2013). The intervention intensity and spacing revealed PFA being delivered ranging from one single session lasting less than 1 hr (Despeaux et al., 2019; Everly et al., 2016; Farchi et al., 2018; Figueroa et al., 2022) to multiple sessions on a weekly or monthly base which spanned 2 weeks to 10 months (Biggs et al., 2016; Ironson et al., 2021; McCart et al., 2020; Ramirez et al., 2013).

#### Tailoring: Intervention Adaptation

The majority of studies did not provide a comprehensive account of how interventions were tailored to specific populations or contexts. Six studies reported some details of their adaptations (Farchi et al., 2018; Hechanova et al., 2015, 2019; McCart et al., 2020; Meir et al., 2012; Ramirez et al., 2013). For example, Hechanova et al. included cultural practices and beliefs in their PFA intervention by encouraging survivors in the Philippines to sing religious songs as a group activity after a typhoon. Meir et al. (2012) also adapted their intervention for Israeli children who were threatened with deportation by using age-appropriate language and being mindful of the local context.

#### How Well: Attrition and Adherence to Protocol

Six studies provided information on attrition rates and protocol adherence. Attendance varied considerably between 41% to 89% (Biggs et al., 2016; Figueroa et al., 2022; Ironson et al., 2021; Ramirez et al., 2013, 2021), with the highest drop-out rate of 89% reported by a study sampling individuals displaced by the Philippines conflict (Mueller et al., 2011). Very few studies documented their adherence to the intervention protocol. Of the two studies that did, one reported a failure to implement the full protocol (Ironson et al., 2021), and a second reported differences in implementation between sites (McCart et al., 2020).

### Synthesis 4: Recipients/Providers’ Perceptions Toward PFA Interventions

#### Perceived Benefits of PFA

Recipients from six qualitative studies reported several psychosocial benefits of receiving PFA, including decreased anxiety, improved self-control, enhanced life functioning, and increased social connectedness (Blake et al., 2021; de Freitas Girardi et al., 2020; Geoffrion et al., 2023; Horn et al., 2019; Schafer et al., 2015; Tessier et al., 2022). PFA was described as a supportive and effective way of enabling individuals to receive time-sensitive assistance when needed after trauma (Geoffrion et al., 2023; Tessier et al., 2022). Providers working in mass trauma situations emphasized the role of PFA in facilitating stepped psychosocial care, reducing mental health stigma, and increasing help-seeking behavior. They also suggested potential long-term benefits that should be further evaluated (Geoffrion et al., 2023; Schafer et al., 2015; Tessier et al., 2022).

#### Satisfaction with PFA Interventions

PFA recipients expressed high levels of satisfaction, reporting that they felt listened to and that their concerns were adequately addressed by providers (Blake et al., 2021). Some also appreciated the practical assistance provided through personalized problem-solving (Hechanova et al., 2015, 2019). The use of group or peer delivery formats was particularly valued, especially among individuals from collectivist and interdependent cultural backgrounds, as it facilitated a shared experience and reduced stigma (Geoffrion et al., 2023; Hechanova et al., 2015, 2019; Tessier et al., 2022). This was also evident in studies using PFA during the COVID-19 pandemic and in emergency medical services, where selecting peers as providers allowed for enhanced trust and openness, leading to a more efficient return to work (Blake et al., 2021; Geoffrion et al., 2023; Tessier et al., 2022).

#### Concerns About Delivering PFA

Non-specialist providers expressed repeated concerns about their ability to engage with individuals who suffered trauma exposure and difficulty in identifying complex mental health needs. They also found it challenging to maintain fidelity to all PFA components, which they suggested diluted intervention delivery and offered false hope or reassurance (Horn et al., 2019; Legerski et al., 2012; Schafer et al., 2015). Providers blamed these problems on inadequate training and lack of intervention tailoring to the population and context (de Freitas Girardi et al., 2020; Horn et al., 2019; Legerski et al., 2012; Schafer et al., 2015). Practical barriers such as limited organization support, restricted time, and limited space or facilities appeared to exacerbate this situation (de Freitas Girardi et al., 2020; Geoffrion et al., 2023; Legerski et al., 2012; Schafer et al., 2015). Emotional burden and secondary traumatic stress were also raised as an issue by providers, especially by non-specialists working with traumatized individuals.

## Discussion

PFA is being increasingly adopted as an early intervention approach to support people affected by trauma. This review examined the evidence for the effectiveness of PFA interventions in improving traumatic stress symptoms and adaptive functioning for individuals after a traumatic event. The way that interventions have been designed, delivered, and experienced both by individuals and providers was also examined. A summary of the critical findings from this review relating to each research objective can be found in Table 3.

### Current Evidence for PFA Intervention Effect

Findings from this review indicate that PFA interventions have been shown to alleviate symptoms of anxiety for individuals exposed to traumatic events across all study designs. In particular, when delivered to individuals exposed to general trauma, such as physical injury, crime, and displacement, PFA interventions lead to a greater reduction in alleviating symptoms of anxiety compared to psycho-education and usual care. While most studies of PFA for preventing PTSD/depressive symptoms produced small but significant effects for symptom reduction in short-term follow-ups, there is less evidence for intermediate or sustained effects on PTSD symptoms. Furthermore, trials of PFA have showed positive improvements in adaptive functioning outcomes, using quality of life and coping measures, while pre-post studies conducted in mass trauma settings found positive effects of PFA on measures of resilience and self-efficacy. Additional potential longer-term benefits of PFA noted by providers included reducing stigma, increasing help-seeking behavior, and self-referral for mental health care in individuals. The identification and validation of these outcome measures, both through qualitative findings and empirical research, similar to the earlier recommendations of selecting sensitive outcomes to evaluate effectiveness aligned with the core goals of PFA (Fox et al., 2012; Vernberg et al., 2008), offer a promising starting point for refining the evaluation methodology and more accurately measuring PFA in any future studies.

This review shows the exploration of PFA effectiveness evaluation has extended beyond mass trauma settings to include general trauma incidents, such as displacement, crime, and physical injuries. This shift from chaotic and varied mass trauma settings to more controlled environments is encouraging, potentially enabling the use of more proactive and rigorous research designs to address current methodological weaknesses found in trials. These limitations include intangible inclusion criteria without assessing trauma antecedents, largely varied sample sizes, inadequate follow-up, and over-reliance on self-reported measures. As ongoing research explores PFA as a support tool for those experiencing other traumatic events such as intimate partner violence, second victims, and other critical incidents (Gispen & Wu, 2018; Ozeke et al., 2019; Stewart et al., 2016), it is likely that a more robust empirical base for PFA efficacy will be established over time.

Referring to the notion that PFA could serve as a selective prevention intervention for individuals at higher risk of developing mental disorders (Tol et al., 2015), the findings from this review only partly support this notion. While a reduction in traumatic stress symptoms has been demonstrated, this does not necessarily prevent PTSD. Although promising results in producing similar effects to established PTSD therapeutic interventions such as EMDR and SMT, these initial findings require further replication to precisely determine the therapeutic potential for PFA.

### Components and Techniques Commonalities Devised Across PFA Interventions

The synthesis of these evaluated PFA intervention packages has revealed several commonalities in the components and techniques used across different PFA approaches. Specifically, these interventions tend to align with four of Hobfoll’s five essential elements, including safety, calm, efficacy, and connectedness (as reflected among 7/11 PFA protocols), whereas the “hope” element was less developed, potentially due to challenges in operationalizing it effectively. This alignment with key elements is further reflected in commonly emphasized components or techniques to achieve these principle elements, including active listening, relaxation/stabilization, problem-solving/practical assistance, and social connection (found in 11 PFA protocols).

Notably, PFA interventions have evolved to become more intensive and complex. This evolution, which includes the incorporation of cognitive reconstruction from established CBT, can be understood in the sense of addressing trauma-related symptoms more effectively. The adaptable nature of PFA to be individually tailored rather than universally applied has been seen as a strength considering the magnitude of trauma and complex situations that individuals may face in aftermath of trauma exposure (Everly & Lating, 2021).

However, adapting how PFA interventions are implemented and combining PFA interventions with other therapeutic interventions for trauma-related symptoms leads to difficulties in evaluating the effectiveness of PFA. The necessity and extent to which PFA intervention components should incorporate with related tactical techniques has not been significantly addressed in the literature. There is a pressing need for further discussion to determine the optimal level of technique integration and to clarify whether PFA, with its evolving complexity and adaptability, still falls within the boundaries of what constitutes PFA or if it has shifted into a different form of intervention altogether.

### Implementation Challenges of PFA Intervention in the Field

In the realm of PFA intervention implementation within trauma contexts, current evaluated PFA interventions are predominately face-to-face, either individually or in groups. Qualitative findings emphasize the perception of PFA as valuable, seen as a time-sensitive, supportive, and practical approach. Shorter single-session PFA are often provided by non-specialist providers, while more intensive multi-session PFA are delivered by mental health specialists. Recipients, particularly in interdependent cultures, prefer peer providers and group formats, highlighting the importance of reducing stigma, and promoting help-seeking behavior. Despite the widespread acceptance of PFA due to its simplicity, timely response, and adaptability, it is imperative to recognize that providers have also reported significant implementation challenges.

The timing and duration of PFA delivery varied considerably, from providing PFA only in the immediate aftermath of trauma exposure to extending it for up to 2 years thereafter, with sessions ranging from one single session to multiple sessions lasting from 2 weeks to 10 months. While guidelines offer limited recommendations, there is a general view for early intervention to be delivered in the initial hours, days, or weeks after trauma exposure (Schnyder & Cloitre, 2022). Shorter PFA delivered early after trauma does indeed fulfill the brief principle of providing immediate support following trauma exposure. However, adding multiple sessions over relatively extended periods merits careful consideration. From one aspect, if accepted, PFA initially originated from crisis intervention focusing on immediacy and short duration to offer rapid access for helping adaptation during the active crisis state of post-trauma. It appears more suitable to view PFA either as a standalone intervention for immediate support or as a component incorporated into a prevention and treatment package or the stepped-care model. As seen in dealing with mental health crisis during the COVID-19 pandemic, PFA in most strategies only provides the first step to facilitate phased mental health care rather than a standalone intervention (Shah et al., 2020; West et al., 2021). From the other aspect, PFA aims to reduce immediate stress and facilitate adaptive functioning, as reflected in its intervention components such as efficacy, connectedness, and hope, delivering PFA sessions within multiple waves may signify an emphasis on maintaining longer-term support and connection to facilitate recovery. However, as argued earlier regarding the optimal intervention components or techniques integrated into the PFA model, there is a risk of blurring the boundary between PFA and more specialized therapeutic interventions or the stepped-care model. In recognizing the fluctuating nature of post-trauma reactions, it is probably not appropriate to define the exact timing being delivered as early versus later phase or the duration as single versus multiple sessions following trauma exposure. Therefore, addressing the challenge of reporting PFA implementation while maintaining a clear distinction from other established interventions becomes an urgent issue.

Regrettably, the current lack of transparency in documenting PFA intervention protocols, particularly regarding their adaptation and fidelity, fails to provide a clear distinction between the diverse nature of PFA. Ideally, PFA is designed to be flexible with the remaining openness to select techniques, allowing its adaptability to be tailored to individuals. Given its adaptability, modifications made to PFA are a reality in implementation. However, this remains an implicit part of the existence of many PFA variants with accompanying manuals (Ni et al., 2023; Wang et al., 2021). This unspoken portion affects the ease of teaching, acquisition, retention, and especially the in-the-field implementation of the corresponding knowledge and skills among providers (Shultz et al., 2014).

This is concerning for non-specialist, first-line lay providers, who are typically the initial point in responding to traumatic exposure and the only contact of care in some situations where human resources and organizational services may be limited (Jordans & Kohrt, 2020). They report difficulties in engaging with individuals who suffered trauma exposure, identifying complex mental health needs, and the PFA delivery is diluted. Moreover, most current PFA training initiatives delivered with minimal teaching often remain unexamined in published research, resulting in varying levels of quality. For instance, a qualitative study investigating one-day PFA training delivered for primary health workers in response to Ebola has reported the danger of making false promises and a diluted PFA delivery, which partly contradicts the fundamental “Do No Harm” approach embodied in the principles of PFA (Horn et al., 2019; Wang et al., 2021). Given this, appropriate training illustrating the significance of cultural competence in delivering PFA, that is, a comprehensive understanding of the complex and dynamic nature of trauma contexts, population needs, and secondary traumatic stress, cannot be overlooked (Wiltsey Stirman, 2022).

While before the training, to address these multifaceted challenges for PFA implementation and evaluation, there is a pressing need for implementation science to address issues related to adaptation, evaluation, and process indicators in refining the PFA intervention prototype for specific populations (Craig et al., 2008; Moore et al., 2021). Ensuring the optimal PFA prototype documented in specific context before scaling up is crucial, which will help the development of better-documented best practices, ultimately enhancing the delivery of PFA and its positive impact on individuals exposed to trauma.

### Strengths and Limitations

This review applied a systematic search and staged synthesis method to critically appraise the published research on PFA. A summary of the findings of this review, along with evidence-based recommendations for research, policy, and practice can be found in Table 4. However, some limitations also need to be considered in this study. Firstly, given the varied PFA description and diversity of PFA approaches, it is possible that relevant studies were not identified. Secondly, as only studies in English and Chinese were reviewed, relevant studies in other languages may have been omitted. Thirdly, the current evidence regarding variability in format, timing, duration, trauma antecedents, and populations receiving PFA and the absence of fidelity data and exclusive reliance on self-report for outcomes bring challenges to determine optimal components and the true impact of PFA.

**Table 4.** Implications for PFA Research, Practice and Policy.

Area	Implications
Research	• Greater transparency and clarity for PFA intervention adaptation, implementation, and evaluation need to be documented. • Rigorous experimental studies to replicate findings in larger samples are suggested, use standard tools with tangible inclusion criteria, and ensure adequate follow-up, which could help explore the selective prevention or therapeutic use for PFA. • Formative research using a participatory method and controlled longitudinal dismantling studies of multi-component interventions with process evaluation embedded are needed.
Practice	• Commonalities such as active listening, relaxation, problem-solving, and social connection, despite their varying sequencing, could potentially be considered certain intervention elements. • Peer provider and group formats can be considered in interdependent cultures and organizational contexts. Especially for organizations that are frequently exposed to traumatic events, multi-phased implementation of PFA can be considered and also explore organizational impact and longer-term benefits. • Organizations should recognize the need for adequate training, supervision, and fidelity monitoring, which is essential to ensure workers deliver PFA with flexibility and precision.
Policy	• Policy-makers should drive consensus building to define what is PFA and what is not, and determine the distinctiveness between prevention and treatment packages. • Efforts to understand how best to deliver PFA and update evidence reviews are needed to guide the optimal PFA implementation. • Collaboration on training and supervising PFA lay providers should be encouraged by international organizations and stakeholders.

*Note.* PFA = Psychological First Aid.

## Conclusion

PFA intervention following trauma exposure shows positive effects for reducing anxiety and facilitating adaptive functioning in the immediate and intermediate term; yet, evidence for reducing PTSD/depressive symptoms is less compelling. Component commonalities or techniques across these PFA interventions include active listening, relaxation/stabilization, problem-solving/practical assistance, and social connection/referral; while cognitive reappraisal has also been incorporated, intensifying PFA. It is important to note that these are not evidence-based components but simply different components or techniques that are subsumed within PFA. More importantly, the significant variation observed in PFA format, timing, and duration, coupled with inadequate documentation of fidelity of implementation and adaptation, limits the potential to inform better practice for PFA intervention delivery.
